# The Effect of Implantoplasty on the Fatigue Behavior and Corrosion Resistance in Titanium Dental Implants

**DOI:** 10.3390/ma17122944

**Published:** 2024-06-15

**Authors:** Darcio Fonseca, Beatriz de Tapia, Ramon Pons, Conrado Aparicio, Fernando Guerra, Ana Messias, Javier Gil

**Affiliations:** 1 Bioengineering Institute of Technology, Medicine and Health Sciences Faculty, Universitat Internacional de Catalunya, Josep Trueta s/n, 08195 Sant Cugat del Vallès, Barcelona, Spain; darciofonseca@beclinique.pt; 2 Department of Periodontology, Faculty of Dentistry, Universitat Internacional de Catalunya, Josep Trueta s/n, 08195 Sant Cugat del Vallès, Barcelona, Spain; b.tapia@uic.es (B.d.T.); mon11@uic.es (R.P.); cjaparicio@uic.es (C.A.); 3 Department Medicina Dentaire, Facultade de Medicina, Universidade de Coimbra, Palácio dos Grilos, Rua da Ilha, 3000-214 Coimbra, Portugal; fguerra@ci.uc.pt (F.G.); amessias@ci.uc.pt (A.M.)

**Keywords:** implantopalsty, fatigue, mechanical properties, titanium, corrosion resistance, ion release

## Abstract

Implantoplasty is a technique increasingly used to remove the biofilm that causes peri-implantitis on dental implants. This technique of mechanization of the titanium surface makes it possible to eliminate bacterial colonies, but it can generate variations in the properties of the implant. These variations, especially those in fatigue resistance and electrochemical corrosion behavior, have not been studied much. In this work, fatigue tests were performed on 60 dental implants without implantoplasty, namely 30 in air and 30 in Hank’s solution at 37 °C, and 60 with implatoplasty, namely 30 in air and 30 in Hank’s solution at 37 °C, using triaxial tension–compression and torsion stresses simulating human chewing. Mechanical tests were performed with a Bionix servo-hydraulic testing machine and fracture surfaces were studied by scanning electron microcopyElectrochemical corrosion tests were performed on 20 dental implants to determine the corrosion potentials and corrosion intensity for control implants and implantoplasty implants. Studies of titanium ion release to the physiological medium were carried out for each type of dental implants by Inductively Coupled-Plasma Mass Spectrometry at different immersion times at 37 °C. The results show a loss of fatigue caused by the implantoplasty of 30%, observing that the nucleation points of the cracks are in the areas of high deformation in the areas of the implant neck where the mechanization produced in the treatment of the implantoplasty causes an exaltation of fatigue cracks. It has been observed that tests performed in Hank’s solution reduce the fatigue life due to the incorporation of hydrogen in the titanium causing the formation of hydrides that embrittle the dental implant. Likewise, the implantoplasty causes a reduction of the corrosion resistance with some pitting on the machined surface. Ion release analyses are slightly higher in the implantoplasted samples but do not show statistically significant differences. It has been observed that the physiological environment reduces the fatigue life of the implants due to the penetration of hydrogen into the titanium forming titanium hydrides which embrittle the implant. These results should be taken into account by clinicians to determine the convenience of performing a treatment such as implantoplasty that reduces the mechanical behavior and increases the chemical degradation of the titanium dental implant.

## 1. Introduction

Peri-implantitis is a biofilm-associated pathological condition characterized by inflammation of the peri-implant mucosa and subsequent progressive loss of supporting bone, is defined as a plaque-associated pathological condition [1] with multiple local-, systemic-, host- and also implant-related factors playing a role in its development and progression.

Dental implants can be made of zirconia or titanium, however the most widely used are the latter. Implants can present different shapes (conical, parallel walls, different thread morphology, etc.) however all of them should be osseointegrated to be functional. A relevant factor to take into account is the surface treatment of the implants that makes them smooth, moderately rough or very rough. Research emerged with the aim of reducing osseointegration times has given rise to increasingly rougher implant surfaces. Once exposed to the oral environment, rough surfaces tend to accumulate more plaque, due to their larger surface area and the protection it provides to bacterial communities against autoclysis. Furthermore, initial bacterial adhesion begins in areas of high wettability, within the grooves of rough surfaces, where it is difficult to remove, resulting in rapid biofilm growth by multiplication [2]. This fact directly influences the progression of the disease, Albouy and coworkers [3,4] observed that the spontaneous progression of peri-implantitis was significantly higher when the roughness of the implant surface increased. There is also evidence that the roughness of the implant surface can play a relevant role not only in the progression of peri-implantitis, but also in the outcome of treatment. Those rougher surfaces had significantly less possibilities of disease resolution after treatment when compared with smoother ones [5,6,7]. 

For this reason, some authors have proposed smoothing and polishing the exposed surface of the implant (implantoplasty) within the treatment of peri-implantitis, not only with the aim of eliminating surface contamination, but also to reduce future bacterial colonization. The effect of implantoplasty in the decrease of plaque adherence and biofilm formation has been corroborated by Azzola and coworkers in 2020 [8], this study demonstrated that implantoplasty significantly produces less growth of biofilm and less mature biofilm as compared to untreated implants. So, when firsts attempt by means of non-surgical treatment have not been enough to obtain disease resolution, which means there is presence of inflammation, bleeding on probing and deep probing pocket depths and there is a need to go through surgical treatment. Whenever implant threads are exposed supra- or subgingivally associated to horizontal bone loss, this technique is frequently included as part of its surgical treatment [9]. 

In some particular clinical conditions, such as peri-implantitis with suprabony defects and superficial infrabony defects [10] in non-esthetic regions, some authors suggest resective surgical therapy with ostectomy and osteoplasty, decontamination of the implant surface, polishing of the supracrestal part of the implant by means of implantoplasty [11] and apically positioned flaps [12]. The objectives in the treatment of these defects are to achieve the reduction of the peri-implant pocket, eliminate peri-implantogenic agents and improve the morphology of the tissues in order to enhance oral hygiene and, consequently, peri-implant health.

**Figure 1:**
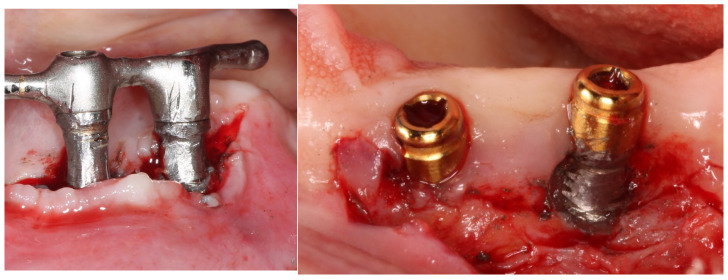
Implantoplasty realized in different dental implants.

To perform implantoplasty, there are different protocols described in the literature, and subsequent to the elimination of the threads by means of diamond burs the polish can be made by Arkansas or silicon burs [13]. However, it has been observed the procedure with the highest capability to reduce roughness (Sa values below 0.1 μm) is the one that uses a sequence of diamond burs with decreasing roughness (106-, 40- and 15-µm) Arkansas burs and silicone polishers [14] The applied protocol eliminates the threads, but tries to leave the diameter of the internal body of the implant, as well as its neck, intact. Figure 1 shows examples of implantoplasty in titanium dental implants. 

With the use of this protocol, it has been described that it is possible to decrease a Ra = 1.94 (standard deviation—SD −0.47) µm to a Ra = 0.39 (SD 0.13) µm. When silicone polishers are used, the roughness values can be reduced up to a Ra = 0.32 (SD 0.14) µm [14] and even Sa values (3D average roughness) of 0.10 (SD 0.02) µm can be obtained [15,16,17].

Furthermore, it has been suggested that this surface modifications could potentiallyimprove the soft tissue adaptation. Recently it has been reported that chairside implantoplasty has the capacity to influence subsequent in vitro fibroblast growth and adhesion. This indicates that implantoplasty treatment outcomes may affect soft tissue healing, adaptation and homeostasis, and that the benefits of its use are not only related to the reduction of microbial colonization [18].

Several authors have proposed that the modification of the implant surface through implantoplasty, together with a resective surgical approach, is an effective way to treat peri-implantitis, since there is an improvement in the clinical parameters of depth of the peri-implant pocket, suppuration and bleeding on probing. Romeo and coworkers [19] in a randomized clinical trial compared two treatment groups, a study group who received a resective surgery and implantoplasty, and a control group in which only resective surgery was performed. The study sample consisted of 17 patients with 35 implants and were followed for 36 months. Three-year results in the study group demonstrated a 100% survival and success rate, with no radiographic bone level changes. On the contrary, in the control group, two implants were lost after two years, decreasing survival to 87.5%, and after three years there had been a mean radiographic bone loss of 1.4 mm mesial and 1.5 mm distally. Clinical benefits associated with the use of implantoplasty as an adjunct to surgical therapy have been described also recently and positive results in terms of disease resolution and implant survival rate have been reported [20]. However, the results after treatment appear to be strongly influenced by the systemic conditions of the patients, oral hygiene habits, configuration of the defect, surface characteristics of the implants, decontamination procedures and the maintenance program to which the patient is included. patients after the intervention [21,22].

Nevertheless, some concern arises regarding the use of this technique. On one hand, the possible impact of the procedure on the mechanical properties of the implant is questioned. Even the intention is not to decrease the internal body size of the implant, it is difficult in practice. Costa-Berenguer and coworkers [14] demonstrated in their study that implantoplasty can cause a slight decrease in the inner diameter of the implant but does not seem to significantly alter fracture resistance of standard diameter implants. Gehrke and coworkers [23] observed a 32% reduction in implant resistance after the implantoplasty procedure was performed. This study suggested that implant wear reduces the resistance to external forces during the application of nonaxial loading. They considered it as a consequence of the change in the architecture of implant-abutment interface which alters the clinical performance and fracture resistance of the implant system after implantoplasty.

On the other hand, implantoplasty leads to the release of titanium debris in enormous quantities to the peri-implant tissues, and this may have adverse biological effects. It has been suggested that this debris can increase the concentration of key inflammatory cytokines that stimulate osteoclasts, and it has been proved to be a contributor to osteolysis [24]. Cell viability assays showed that these particles produce a significant loss of cytocompatibility on osteoblasts and fibroblasts, which means that the main cells of the peri-implant tissues might be injured [15,16]. A recent in vitro study demonstrated that performing implantoplasty on dental implants produces debris mostly within the ultrafine size range (<100 nm) [25]. This particle size is within the range of debris capable of exerting high levels of biological and immunological activity. These ultrafine debris are biologically more detrimental compared to the visible coarse particles, through biochemical mediators of inflammation, cellular recruitment, and bone resorption [26]. In this regard, its recommended to consider procedures trying to reduce the widespread release of debris and particles generated during implantoplasty as could be the use of a rubber dam or high-volume evacuation. 

Implantoplasty can be an ally to improve the local clinical conditions of the implant, however its application is not free of danger and complications, and there is need to improve the knowledge on how it could affect the viability and the biological behavior of dental implants, so the aim of the present study is to evaluate the effect of implantoplasty on the fatigue behavior and corrosion resistance in titanium dental implants.

## 2. Materials and Methods

### 2.1. Sample Preparation and Collection

**Figure 2:**
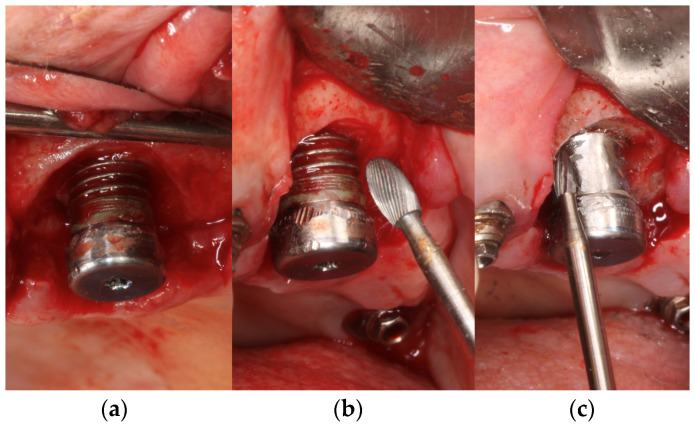

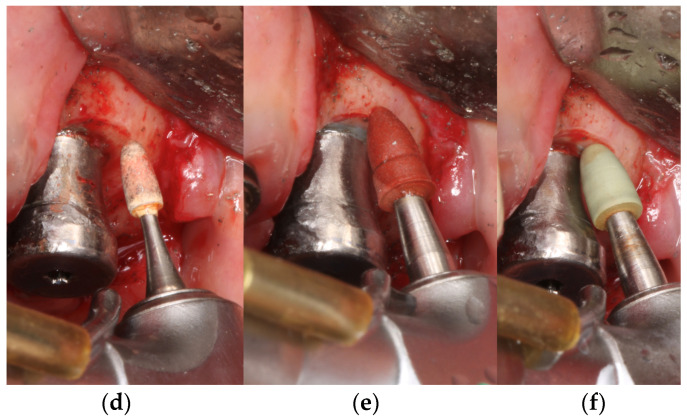
Drilling protocol used in the implantoplasty process. (**a**) Modification of the surface with a fine-grained tungsten carbide bur. (**b**) Larger burs employed on the vestibular and palatal sides. (**c**) Smaller diameter burs utilized in areas with limited access or interproximal spaces. (**d**) Refined finish treatment with coarse-grained. (**e**) using medium-grained drills. (**f**) using fine-grained drills.

Implantoplasty of commercially titanium grade 3 dental implants (Klockner Implant System, Escaldes-Engordany, Andorra) was carried out by the same investigator (D.F) following the following drilling protocol. Using a GENTLEsilence LUX 8000B turbine (KaVo Dental GmbH, Biberach an der Riß, Germany) under constant irrigation, the surface was sequentially modified with a fine-grained tungsten carbide bur (reference H379.314. 014 KOMET; GmbH & Co. KG, Lemgo, Germany) (Figure 2a). Tungsten carbide burs serve as the primary tool for the initial shaping of implant prostheses, with the size of the bur tailored to the specific area being treated. Typically, larger burs are employed on the vestibular and palatal sides (Figure 2b), while smaller diameter burs are utilized in areas with limited access or interproximal spaces (Figure 2c). These burs effectively remove implant threads, ensuring a smooth surface texture. Additionally, to achieve a refined finish, a series of polishing drills are employed, progressing from coarse-grained to fine-grained drills (Figure 2d–f). A coarse-grained silicon carbide polisher (order no. 9608.314.030 KOMET; GmbH & Co. KG, Lemgo, Germany) and a fine-grained silicon carbide polisher (order no. 9618.314.030 KOMET; GmbH & Co. KG, Lemgo, Germany) [14].

**Figure 3:**
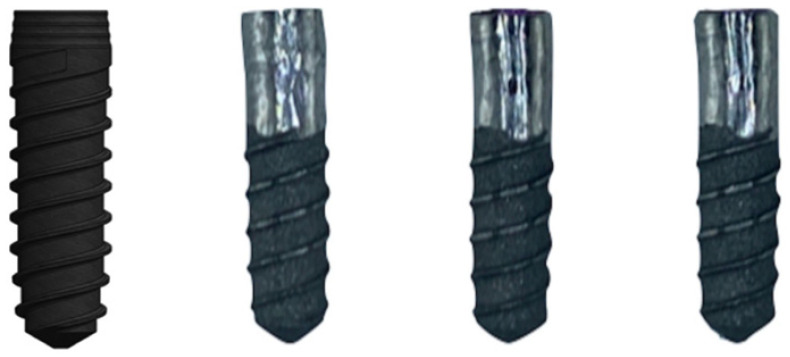
As-received dental implant and three examples of the implant treated by implantoplasty.

The dental implants control and after of the implantoplasty procedure can be observed in Figure 3. 

### 2.2. Scanning Electron Microscopy

Morphology was investigated using a Jeol 6400 scanning electron microscope (JEOL, Tokyo, Japan) with a resolution of 15 nm and an acceleration voltage of 20 keV. Gold coating of the surfaces by sputtering was not necessary as the samples were sufficiently conductive. In addition, the microscope was coupled with an energy-dispersive X-ray microanalysis system (EDS Oxford, Oxford, UK). Analysis of images software (ImageJ) version 3.2. (Java, Silicon Valley, CA, USA) was used in order to determine the measures of the different phases or pitting produced [27].

### 2.3. Mechanical Characterization

Mechanical behavior was tested by using a servo hydraulic mechanical testing machine BIONIX 370 (MTS, Minneapolis, MN, USA) equipped with a 25 kN load cell controlled by software Telstar II version 2.0 (Telstar, MTS System Corp., Eden Prairie, MN, USA). One hundred twenty implants were tested at 25 °C under dry conditions (n = 30) and in Hank’s solution at 37 °C (n = 30) for control and treated by implantoplasty. The chemical composition of Hank’s solution (ThermoFisher, Madrid, Spain) is shown in Table 1 [28,29,30].

**Figure 4:**
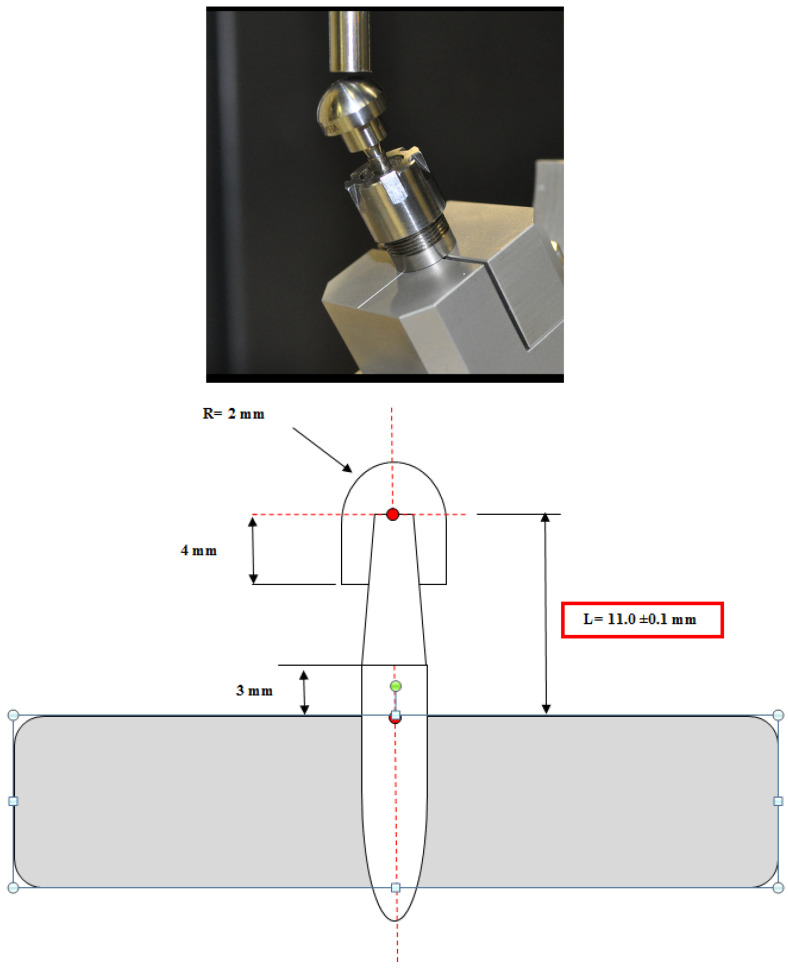
Mechanical testing machine and the dimension of the samples. The angle used was 30°.

Five specimens for each of the treatments studied were statically tested to determine the maximum breaking load. The jaws shown in Figure 4 were made of stainless steel with an angle of 30°, in the scheme can be seen the lengths and characteristics of the mechanical test. The load application rate was 1 mm/min and was performed at the distal cusp of the abutment [31,32,33]. 

Fatigue tests were performed between 10% and 80% of the maximum value of the ultimate strength for each group [30]. In all cases, 5 specimens per treatment were performed. The mechanical cycles were sinusoidal in compression with a frequency of 15 Hz. The total number of cycles was set at 10 × 10^6^ [31,32,33]. 

### 2.4. Corrosion Resistance

For the corrosion tests, 30 samples were used, 10 samples per treatment. The test surface for all samples was 19.6 mm^2^. The electrolyte used was Hank’s solution whose chemical composition can be seen in Table 1. 

**Figure 5:**
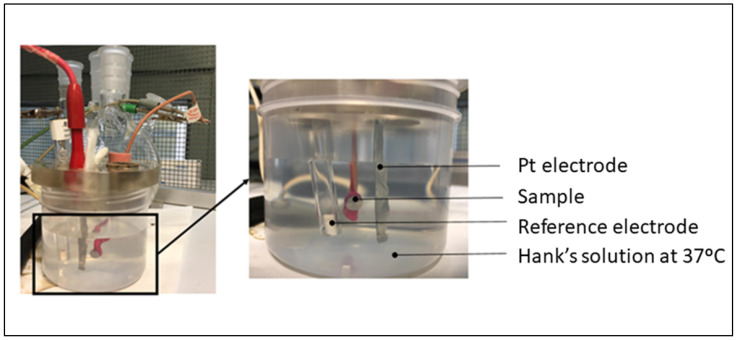
Experimental set up used for assessing corrosion resistance.

The reactor was a 185 mL polypropylene (PP) tank closed with a polymethyl methacrylate plug with different holes for the introduction of the sample, the reference electrode, and the counter electrode. The test scheme can be seen in Figure 5. The calomel electrode (saturated KCl) was used as a reference in all tests, with a potential of 0.241 V compared to the standard hydrogen electrode. The tests were carried out with the protection of a Faraday cage and at a temperature of 25 °C.

The open circuit potential was performed for 5 h for all samples studied and potentials were measured every 10 s. The potential was considered to be stabilized when the change in potential was less than 2 mV over a period of 30 min, as indicated in ASTM standards [34,35,36,37]. Data and E-t curves were obtained using PowerSuite software (version 3.0) (Deavon-Berwyn, PA, USA) with the PowerCorr-Open circuit. 

Cyclic potentiodynamic polarisation curves were obtained for the 3 study groups following the ASTM G5 standard [35]. In this test, a variable electrical potential is imposed by the potentiostat between the sample and the reference electrode, causing a current to flow between the sample and the counter electrode. The counter electrode used was platinum [38,39]. Before starting the test, the system was allowed to stabilise by means of an open-circuit test for 1 h. After stabilization, the potentiodynamic test was launched, performing a cyclic sweep from −0.8 mV to 1.7 mV at a speed of 2 mV/s. These parameters were entered into the PowerSuite program using the PowerCorr-Cyclic Polarization function to obtain the curves. The parameters studied were:

- icorr (μA/cm^2^)/corrosion current density.
- Ecorr (mV)/Corrosion potential: value at which the current density changes from cathodic to anodic.

In accordance with the ASTM G102-89 standard [36], these values are then used to calculate the polarization resistance (R_p_) and the corrosion rate (CR in mm/year) [37]. The polarisation resistance indicates the resistance of the sample to corrosion when subjected to small variations in potential. 

### 2.5. Ion Release

For the study of the release of titanium ions, 5 samples of each treatment were used following the ISO 10993-12 standard [38]. All samples were weighed with a gram sensitivity balance (Sartorius 2045, Barcelona, Spain). All the solutions were prepared by adjusting 1 mL of Hank’s solution for every 0.20 g of sample, as indicated by the standard [39,40]. Samples were placed in an Eppendorf with 5 mL of Hank’s solution and stored at 37 °C. Hank’s solution should be extracted and stored in the refrigerator after 1 to 12 days. After each extraction, 5 mL of fresh Hank’s solution is refilled into the Eppendorf containing the samples. 

After every day from the first to twelfth, the concentration of released titanium ions was measured by inductively coupled plasma mass spectrometry (ICP-MS) with Agilent Technologies 7800 ICP-MS (Agilent Tech. Santa Clara, CA, US).

### 2.6. Statistical Analysis

The number of samples used was obtained by an experimental sample size method. Statistical analysis was performed using MiniTab 17 software (Minitab Inc., State College, PA, USA). Kruskal-Wallis and Mann Whitney U non-parametric tests were used to compare the different conditions to each other. Statistical differences were considered with *p* < 0.005.

## 3. Results

**Figure 6:**
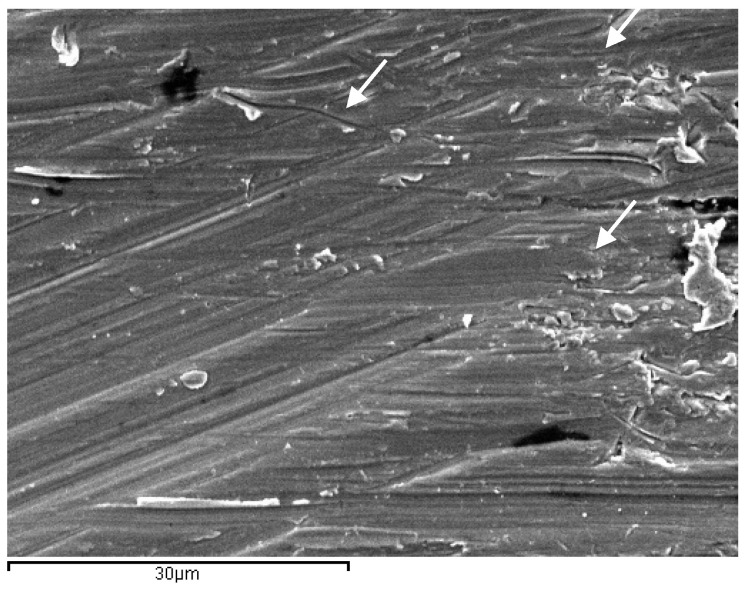
Surface of the dental implant treated by implantoplasty. The arrows show marks and small voids produced by machining process which can nucleate cracks caused by fatigue.

Implantoplasty treatments generate a mechanization of the surface. Figure 6 shows the surface of the implant that has undergone implantoplasty. Small surface defects, indentations and small cracks can be observed, which can facilitate nucleation or the rapid propagation of a fatigue crack.

**Figure 7:**
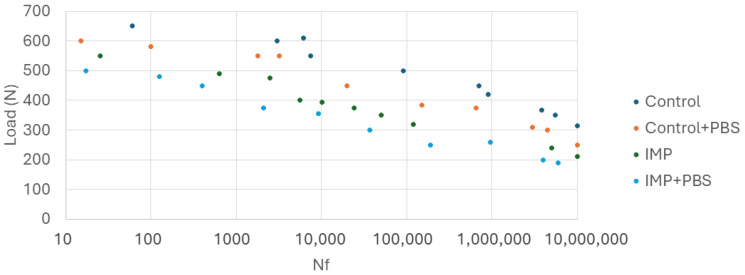
Curves of fatigue for the different dental implants studied. (IMP: implantoplasty and PBS: Physiological Body Simulation by Hank’s solution at 37 °C).

Figure 7 shows the fatigue life curves of control and implantoplasty-treated dental implants. It can be seen that implantoplasty implants have a shorter fatigue life at a given chewing force. It can also be seen in the curve that dental implants treated in Hank’s solution at 37 °C have a shorter fatigue life than those tested dry.

**Figure 8:**
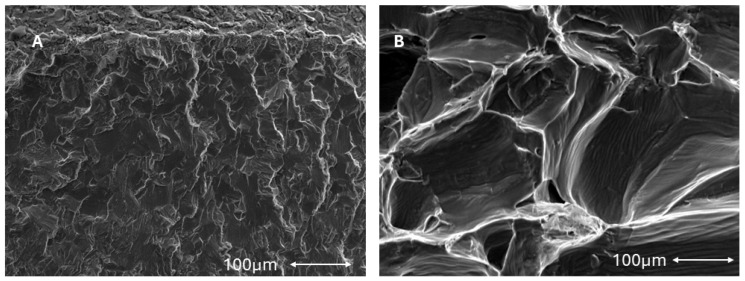
(**A**). Intragranular fracture of the titanium dental implant submitted to fatigue without Hank’s solution (**B**). Intergranular fracture of the titanium dental implant submitted to fatigue without Hank’s solution at 37 °C.

The fractography shows the intragranular fracture for the implants without PBS and intergranular fracture for the implants immersed in Hank’s solution. This fact is due to the appearance of titanium hydrides in the grain boundaries. The fractures can be observed in Figure 8. 

**Figure 9:**
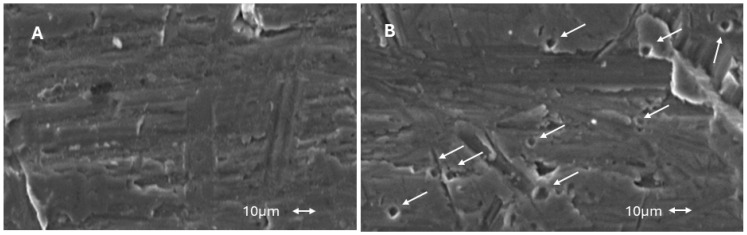
(**A**) Surface of the titanium control after corrosion test (**B**). Samples with implantoplasty treatment after the corrosion test. Arrows indicate the pitting.

Table 2 shows the corrosion results, with higher corrosion values for the dental implants that have undergone the implantoplasty processes. In Figure 9 it can be observed by scanning electron microscopy the surface of the control samples and the implantoplasty samples with presence of pitting on the surface. The defects produced by machining become favorable susceptible points for electrochemical corrosion.

**Figure 10:**
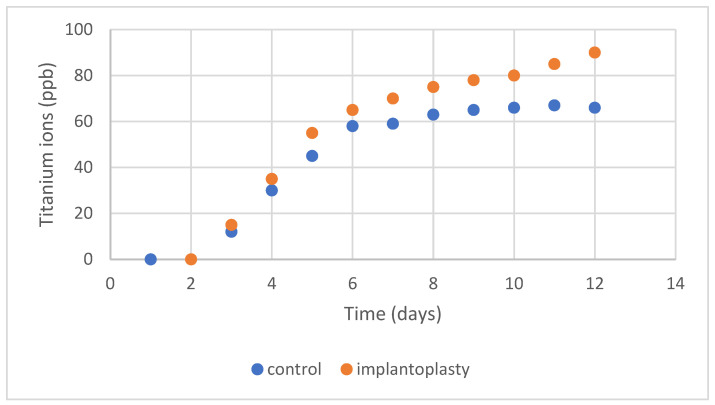
Titanium ion release at different times for surfaces of 1 cm^2^.

The release of titanium ions as a function of immersion time in Hank’s solution is shown in Figure 10. A rapid growth of the ion release can be seen to reach almost constant values from the fifth day onwards. It can also be seen that the values obtained are parts per billion and are therefore very small values. From the figure it can also be seen that the surfaces with implantoplasty show slightly higher release values than the control.

By making an adjustment to obtain a mathematical model, adjustments can be observed following neperian logarithm models for the case of the control samples and the implantoplasty samples were:

For control. [Ti] = −1.9 + 33.2 lnt

For implantoplasty [Ti] = −2.9 + 55.4 lnt

Being [Ti] concentration in ppb of titanium ion release and t is time of immersion in days.

As can be observed, the value of the implantoplasty produces a higher growth of released ions with respect to the immersion time (33.2 for control in relationship with 55.4 for implantoplasty samples). The correlation coefficients for adjustments have been 0.987 for the control and 0.983 for the implantoplasty case.

## 4. Discussion

According to the Health Comission of the European Union, 24% of dental implants present periodontal diseases due to bacterial colonization in 12 years. Of these dental implants colonized by bacteria, 13% end up undergoing implantoplasty. Normally, good clinical practice indicates that the infected dental implant should be removed, the area should be sanitized, sometimes it is necessary to regenerate the bone with bioactive calcium phosphate materials, wait for bone formation and place a new dental implant. This is expensive for the patient, presents a certain clinical complication and takes a long time. Especially, this solution is difficult to accept for old people. In addition, sometimes, the placement of a new implant requires the extraction of a tooth next to the infected implant area to insert a larger diameter implant. These complications make implantoplasty a technique that in recent years is increasing its use to values close to 27% of dental implants with peri-implantitis. This fact is worrying the health authorities since the mechanization of titanium produces a reduction of surface and therefore of static and fatigue mechanical properties as well as resistance to corrosion as we have seen in this research.

However, the degree of toxicity and inflammation caused by the particles left in the mouth because of machining remains to be determined. It is important to carry out studies on this technique so that clinicians can take into consideration the effects that it may have in the long term. Preliminary results by the health authorities are that inflammation occurs in all cases in the area, especially in dental implants made of Ti6Al4V and to a lesser degree in those made of commercially pure titanium. The fracture data of implants favored by implantoplasty are estimated to range from 17 to 25% of those placed in less than 5 years. 

From the in vitro studies of this research, the fatigue results it can be observed that the control dental implants with respect to those that have undergone implantoplasty have a loss of fatigue life in the order of 30% and in physiological environment it increases to a loss of 37%. This fact is due to the fact that implantoplasty, according to the protocol applied to dental implants, reduces the effective surface by 0.6 mm. In addition to this cause there is another factor that causes the implantoplasty which is the plastic deformation that generates the machining on the surface of the implant creating on the surface areas susceptible to nucleation of cracks or sometimes small cracks caused by the drills (Figure 6). These small cracks are the ones that, due to the cyclic loads caused by chewing, propagate to the fracture of the dental implant [32,33,41,42,43,44]. 

Likewise, when we compare the results of fatigue in both types of dry implants with those tested in a physiological environment, we observe a loss of fatigue life for the control implants of 12% and for those treated with implantoplasty of around 18%. This loss in the fatigue life of dental implants is due to the fact that the physiological environment causes corrosion at the crack tip which accelerates its propagation. This fact has been proven in the fatigue of hip prostheses where the fatigue limit in fatigue tests is 770 MPa, in air it drops to 650 MPa and in physiological medium it drops to 550 MPa. Therefore, the values given in the fatigue studies, which the international standard indicates that they are performed in air, must be known that they decrease due to the effect of the physiological medium when the dental implant is in service [45,46,47,48].

In dental implants at bone level, the whole body of the implant presents a roughness obtained by grit-blasting of abrasive alumina particles. The projection of abrasives to obtain a topography favorable to adhesion, proliferation and osteoblastic differentiation also causes a compressive residual stress, which according to the studies of Guillem-Marti and Pereira et al. by means of Bragg-Bentane X-ray diffraction is estimated at −200 MPa of compressive load [49,50]. This compressive residual stress zone disappears with the mechanization of the implantoplasty. This fact makes that the non-mechanized dental implant has residual stress, and the treated one presents an absence of stress, this fact favors that in the zones of change of properties the formation of a crack is favored as well as the zone becomes susceptible to present electrochemical corrosion [51].

It has been observed that in some cases, the equilibrium decomposition of water can cause hydrogen cations to enter the interior of fatigued titanium. These hydrogen atoms migrate by diffusion to the titanium grain boundaries, since these are areas where there is more space and where hydrogen proton bonding occurs to form the hydrogen molecule (H_2_), which has a larger volume. This increase in volume at the grain boundary causes stresses and can generate cracks that fracture the implant with an intergranular morphology in a brittle manner [52,53].

The corrosion potential values and especially the corrosion intensity values show an increase in the implants that have undergone implantoplasty with statistically significant differences. This acceleration of corrosion is due to the fact that the areas that have been machined present stresses that form an anodic zone with respect to the areas that have not been machined that become cathodic zones and pitting can be observed in the areas of behavioral change [54,55]. These pitting can also become crack nucleation zones due to masticatory loads. From the results we can appreciate that titanium implants with implantoplasty increase the corrosion rate to values of 4.57 × 10^−4^ mm/year.

The titanium ion release results have a curve of a rapid increase and then enter a constant release zone, being able to appreciate an increase in the values of higher ions for the samples with implantoplasty. However, the values obtained are relatively low, in the order of ppb, which with respect to other endosseous implants in the human body, such as hip or knee implants, are values of ppm. It is for this reason that the release of ions should not be of excessive concern [56,57,58,59,60].

The results of this research should give clinicians food for thought regarding clinical treatments of implantoplasty to resolve peri-implantitis. Several authors have shown the good clinical results of implantoplasty [61,62,63,64,65,66,67,68,69,70,71,72,73] but as we can observe it presents disadvantages, such as significant reduction of fatigue life, increased corrosion rate among others. One of the dangers that this treatment produces in the patient’s mouth should be studied, which is the release of titanium particles of different sizes that cannot be aspirated. At this point, toxicity analyses should be studied according to the particle sizes. It has been determined that implantoplasties, realized in vitro, performed on dental implants manufactured with Ti6Al4V alloy have a much higher risk of toxicity than those manufactured with commercially pure titanium. The cell survival rates were less than 70%, approximately between 60–65%, which indicates cytotoxicity in the case of commercially pure titanium does not exceed 75% survival [65,67,70]. It has been observed in cellular studies that the response of inflammatory cells is superior in the titanium alloy with respect to titanium. In both materials studied it has been possible to demonstrate the higher degree of toxicity of the particles smaller than micrometer of equivalent diameter since they do not seem to be detected by the immune system. It has been determined that particles between 1 and 5 μm are phagocytosed by macrophages and particles larger than 5 μm form a fibrotic capsule. One of the phenomena studied is that implantoplasty causes inflammation in the patient’s soft tissues and the immune system produces a drop in oxygen content that causes the death of aerobic bacteria. However, the anareobic bacteria are not altered by the mechanism of the lowering of the oxygen content. It has been possible to determine how the oxygen that forms the titanium passivation layer can be reduced to titanium metal, releasing the oxygen that is taken up by the tissues. This reduction of titanium oxide to pure titanium generates mixed oxides when the inflammation decreases and the oxygen content increases, which present toxicity [14,15,16,67,68]. This phenomenon that has been observed makes it necessary to treat titanium that has undergone implantoplasty for its repassivation and to avoid the formation of these non-stoichiometric oxides.

These results should be taken into account by clinicians in determining the appropriateness of implant treatment for the patient they are treating. It is necessary to complete this work with other types of drills and other instruments used that can be used in implantoplasty and to determine the variation of properties with the results of this research. It would be very convenient to have an optimal protocol for the treatment of implantoplasty in order to improve the technique and improve the long-term behavior of the treated implants. In the same sense, it is necessary to obtain a chemical solution that can be applied in vivo on the treated surface of titanium implants in order to reduce the corrosion resistance and the titanium ion release.

Implantoplasty should be used in a very careful way and when there is no possibility of doing another type of treatment or the removal of the dental implant that presents peri-implantitis is unanchored. Likewise, it is still pending to describe a commitment protocol in which, in case of performing this technique, the least possible damage is done to the patient and to the dental implant so that it can continue to fulfill its mission.

## 5. Conclusions

Implantoplasty treatments reduce the fatigue life of titanium dental implants by 30% in air and 37% in physiological medium. Implantoplasty creates two zones, one with plastic deformation that becomes the anodic zone and another non-deformed zone that acts as a cathodic zone causing pitting in the dental implant and reduces the corrosion resistance of the dental implant with implantoplasty. The release of titanium ions to the physiological medium is higher for machined implants although the levels are parts per billion considered non-cytotoxic. 

## Figures and Tables

**Table 1:** Composition of Hank’s solution.

Chemical Product	Composition (mM)
K_2_HPO_4_	0.44
KCl	5.4
CaCl_2_	1.3
Na_2_HPO_4_	0.25
NaCl	137
NaHCO_3_	4.2
MgSO_4_	1.0
C_6_H_12_O_6_	5.5

**Table 2:** Corrosion values of the control and the surfaces machining by the implantoplasty process. Asterisks means the statistical difference significance (*p* < 0.005).

Samples	E_corr_ (mV)	i_corr_ (μA/cm^2^)	R_p_ (Ω/cm^2^)	Corrosion Rate (mm/year)
Control	−302 ± 22	0.041 ± 0.006	$1.22\times 10^{6}\pm 1.03\times 10^{5}$	$4.02\times 10^{-4}\;\pm 7.79\times 10^{-5}$
Implantoplasty	−345 ± 38 *	0.050 ± 0.0085 *	$1.02\times 10^{6}\pm 1.33\times 10^{5}$ *	$4.57\times 10^{-4}\;\pm 3.23\times 10^{-5}$ *

## Data Availability

The original contributions presented in the study are included in the article, further inquiries can be directed to the corresponding author.
